# Translational Theranostics in Glioblastoma: FAPI-46 as a Precision Medicine Tool

**DOI:** 10.2967/jnumed.125.271759

**Published:** 2026-09

**Authors:** Pauline Jeanjean, Abir Swaidan, Christopher Tse, Samantha Kwock, Iveta Fajnorova, Rachel Dove, Ines Camille Azrour, Sarah Taylor, Begonya Comin-Anduix, David A. Nathanson, Giuseppe Carlucci, Jérémie Calais, Johannes Czernin, Élie Besserer-Offroy, Christine E. Mona

**Affiliations:** 1 Ahmanson Translational Theranostics Division, Department of Molecular and Medical Pharmacology, David Geffen School of Medicine, UCLA, Los Angeles, California;; 2 Jonsson Comprehensive Cancer Center, UCLA Health, Los Angeles, California;; 3 Division of Surgical Oncology, Department of Surgery, UCLA, Los Angeles, California; and; 4 California NanoSystems Institute, UCLA, Los Angeles, California

**Keywords:** glioblastoma, fibroblast activation protein, theranostics, targeted radiopharmaceutical therapy, immunomodulation

## Abstract

Glioblastoma represents the most lethal form of brain cancer, characterized by a 5-y survival rate of approximately 4%. Fibroblast activation protein (FAP), expressed within both the tumor microenvironment and glioblastoma cells, has been identified as a promising therapeutic target. This study explores the potential of FAP-targeted molecular probes as theranostic agents for glioblastoma. **Methods:** Human U-87 MG and murine SB28 glioblastoma-derived cell lines were used to establish immunodeficient and immunocompetent models, respectively. FAP inhibitor (FAPI)–46 was radiolabeled with ^68^Ga for imaging and ^225^Ac or ^177^Lu for therapy. Tumor uptake, therapeutic efficacy, immunomodulatory effects, and survival were evaluated for FAPI-46 alone or in combination with temozolomide in preclinical studies. [^68^Ga]Ga-FAPI-46 tumor uptake was also evaluated in patients. **Results:** In subcutaneous xenografts, [^68^Ga]Ga-FAPI-46 exhibited high tumor uptake with minimal background signal. In the immunocompromised model, a single dose of [^225^Ac]Ac-FAPI-46 combined with temozolomide was sufficient to increase median survival, whereas in the immunocompetent model, 3 consecutive daily doses were required to achieve a significant survival benefit. [^177^Lu]Lu-FAPI-46 demonstrated lower therapeutic efficacy, as a monotherapy or in combination with temozolomide, than did [^225^Ac]Ac-FAPI-46. Immunomodulatory effects, assessed via flow cytometry, were insufficient to overcome the immunologically “cold” glioblastoma tumor microenvironment. In orthotopic xenografts and patients, [^68^Ga]Ga-FAPI-46 showed low uptake, indicating limited blood–brain barrier penetration and resulting in tumor concentrations likely too low for therapeutic efficacy. **Conclusion:** FAPI-46 demonstrates significant potential as a theranostic agent for glioblastoma in peripheral models. However, its inability to cross the blood–brain barrier in orthotopic models highlights the need for future studies to improve its clinical applicability.

## Visual Abstract

**Figure:**
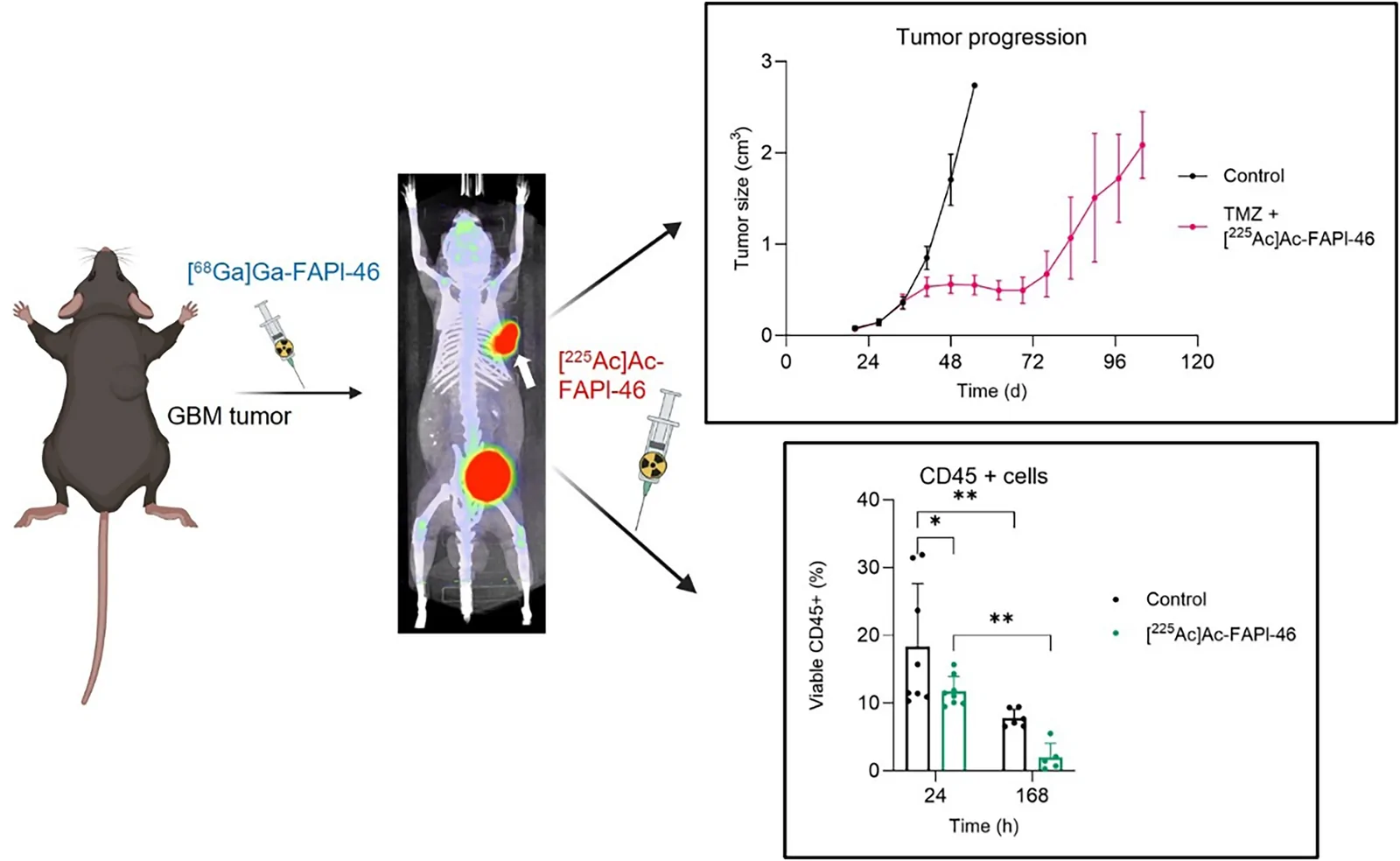

Glioblastoma is an aggressive and highly infiltrative brain tumor with a 5-y survival rate of 4% (1–3). The current standard of care, the Stupp protocol (4,5), which combines surgical resection with chemotherapy and radiotherapy, has proven to be insufficient (6). Hence, significant tumor heterogeneity complicates the development of effective multitargeted therapeutic strategies (7–9). Glioblastoma progression and resistance to therapy are largely driven by complex interactions between the tumor microenvironment (TME) and tumor cells (2,3,7).

A key factor in this progression is fibroblast activation protein (FAP), a type 2 transmembrane glycoprotein, whose overexpression in glioblastoma correlates with poor prognosis (10–12). FAP is expressed on tumor and TME cells, including cancer-associated fibroblasts, endothelial cells, and pericytes, making it an attractive therapeutic and imaging target (10,13–15). FAP-targeting PET imaging ligand potential has been demonstrated in several cancers, including glioblastoma (16–18). Beyond imaging, FAP-targeting radiopharmaceutical therapy (RPT) has shown encouraging activity in sarcoma (19), highlighting a potential avenue for glioblastoma treatment. FAP RPT targets both FAP-expressing tumors and TME cells, potentially enhancing therapeutic efficacy through dual engagement of the tumor and its supporting microenvironment.

In this study, we investigate the use of FAP inhibitor (FAPI)–46 RPT in glioblastoma models overexpressing FAP. Its in vivo efficacy was first evaluated as monotherapy and in combination with temozolomide in subcutaneous xenograft and syngeneic models to confirm therapeutic efficacy. Subsequently, orthotopic models were used to investigate the impact of blood–brain barrier (BBB) limitations on FAPI-46 delivery and therapeutic response, given that an intact BBB represents a major obstacle for central nervous system drug delivery.

## MATERIALS AND METHODS

Materials and methods are provided in the supplemental materials (supplemental materials are available at http://jnm.snmjournals.org).

## RESULTS

### FAP Expression in Patients Is Conserved in Patient-Derived Orthotopic Xenografts (PDOXs)

To evaluate the relevance of FAP as a biomarker in glioblastoma, we examined its expression in patient-derived tumors and models (20). RNA sequencing of tumor cells isolated from the TME of patient-resected glioblastoma revealed FAP overexpression in approximately 25% from the UCLA cohort, relative to normal brain (Supplemental Fig. 1). Comparative analysis with the Cancer Genome Atlas dataset (National Institutes of Health) showed a similar trend, although FAP expression levels were significantly higher in the Cancer Genome Atlas samples (Supplemental Fig. 1). In contrast to the Cancer Genome Atlas dataset, RNA sequencing in the UCLA cohort was performed exclusively on isolated cells, which may account for the lower relative expression levels observed.

To further assess the translational fidelity of FAP expression, immunohistochemistry analysis was performed on orthotopic PDOXs and their matched patient tumors, revealing preserved FAP expression patterns between the original human tumors and the corresponding PDOX models (Supplemental Fig. 1). However, costaining with cell type–specific markers was not performed to attribute FAP expression to tumor versus TME cell populations; therefore, a contribution from TME cells (cancer-associated fibroblasts and pericytes) cannot be excluded in both PDOX and patient samples.

Taken together, these results indicate that FAP is overexpressed by glioblastoma and TME cells and that its overall expression is faithfully conserved in vivo in PDOX glioblastoma models, supporting their use as a relevant preclinical system for FAP-targeted therapy evaluation.

### Combined FAP RPT and Chemotherapy Induces Significant Tumor Growth Delay in a Human Glioblastoma Model

**FIGURE 1.**
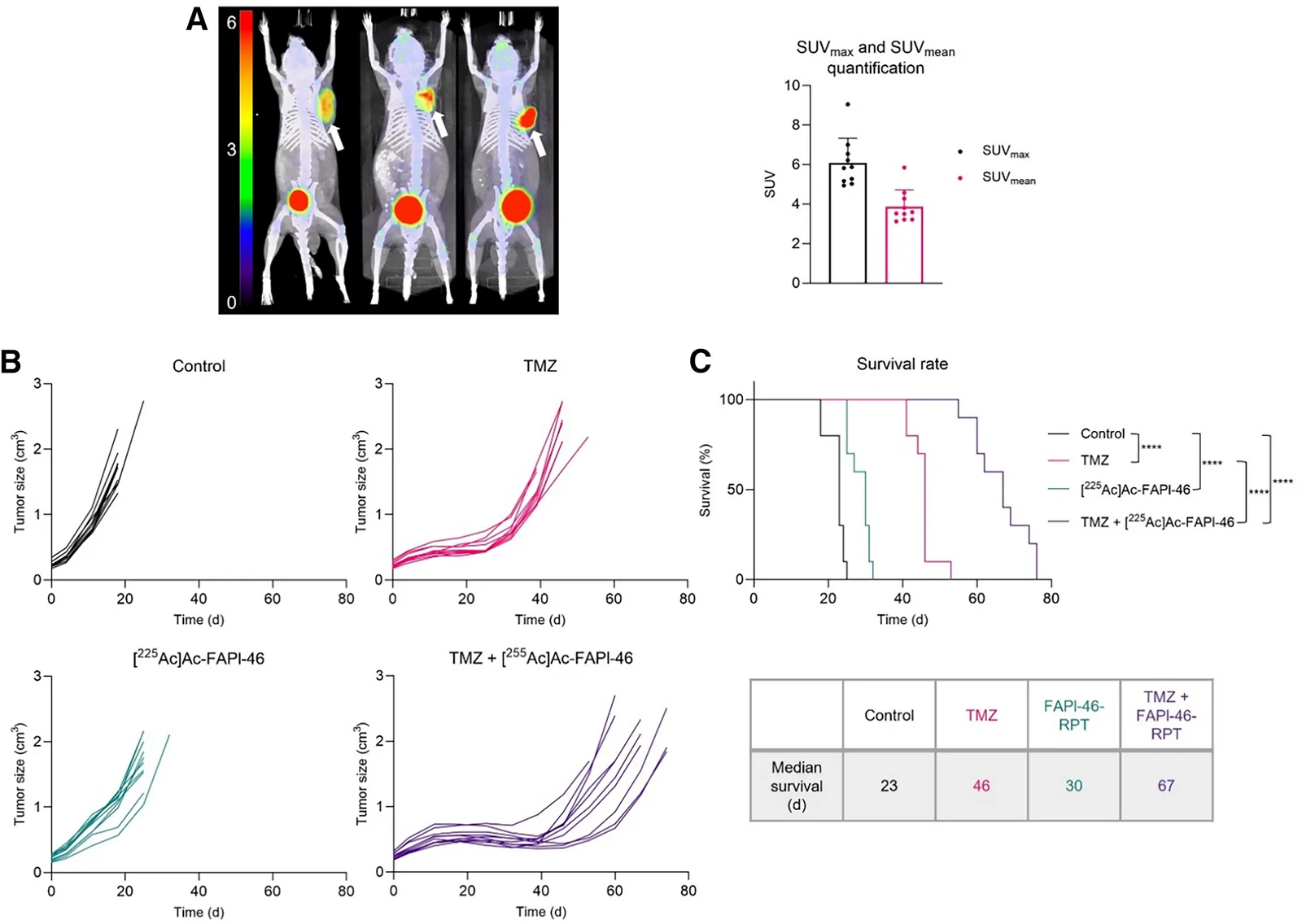
Single-dose [^225^Ac]Ac-FAPI-46 combined with TMZ in subcutaneous U-87 MG model. (A) [^68^Ga]Ga-FAPI-46 PET/CT (left), and SUV_max_ (black) and SUV_mean_ (pink) quantification in U-87 MG subcutaneous tumors (right, *n* = 10). Arrows indicate location of tumor. (B) Tumor progression in control (black), [^225^Ac]Ac-FAPI-46 (green), TMZ (pink), and combination-therapy (purple) groups (*n* = 10/group). (C) Kaplan-Meier curve with median survival per group. Survival curves were compared using log-rank (Mantel–Cox) tests. ****P < 0.0001. TMZ = temozolomide.

U-87 MG, a human-derived glioblastoma cell line displaying high endogenous FAP expression, was used to generate subcutaneous xenografts (21). FAP expression was confirmed both in vitro (75.9% of positive cells) and ex vivo (Supplemental Fig. 2). [^68^Ga]Ga-FAPI-46 PET/CT scans revealed significant tumor uptake, with an average SUV_max_ of 6.1 ± 1.2 and SUV_mean_ of 3.9 ± 0.8 (Fig. 1A).

To assess the relevance of FAP RPT in glioblastoma, we developed a modified version of the standard glioblastoma treatment protocol by substituting external beam radiation with FAPI-46 RPT (4,5). Monotherapies with [^225^Ac]Ac-FAPI-46 or temozolomide resulted in tumor growth delays of 7 and 23 d, respectively, compared with untreated animals. Combination therapy of [^255^Ac]Ac-FAPI-46 and temozolomide yielded a further survival benefit, extending median survival by 21 d beyond temozolomide alone (Figs. 1B and 1C) (19). Consistent with the therapeutic activity of an α-emitter, [^225^Ac]Ac-FAPI-46 induced DNA double-strand-break damage, as evidenced through increased γH2AX phosphorylation 24 h post-treatment. Quantitative analysis revealed a significant increase in γH2AX-positive nuclei (7.0 ± 1.2%) compared with untreated tumors (1.2 ± 0.8%) (Supplemental Fig. 3).

Substitution of the α-emitter radionuclide (^225^Ac) with a β-emitter radionuclide (^177^Lu) showed a delay in tumor growth of 8.5 d compared with the control group (median survival, 16.5 and 25 d, respectively). Two cycles of temozolomide induced a cytostatic effect for around 35 d, followed by a relapse in tumor growth. However, combination therapy did not demonstrate a significant difference in tumor growth compared with temozolomide alone (median survival, 51 and 44 d, respectively) (Supplemental Fig. 4).

Taken together, these results confirm the effective tumor uptake of the [^68^Ga]Ga-FAPI-46 probe in U-87 MG xenografts and demonstrate that α-emitter–radiolabeled FAPI-46 induces double-strand breaks, delays tumor growth, and enhances survival when combined with temozolomide. In contrast, β-emitter–radiolabeled FAPI-46 did not induce a significant therapeutic effect when combined with temozolomide. Therefore, subsequent investigations were focused on [^225^Ac]Ac-FAPI-46.

### Triple-Dose [^255^Ac]Ac-FAP Significantly Increases Median Survival in Immunocompetent Mice

**FIGURE 2.**
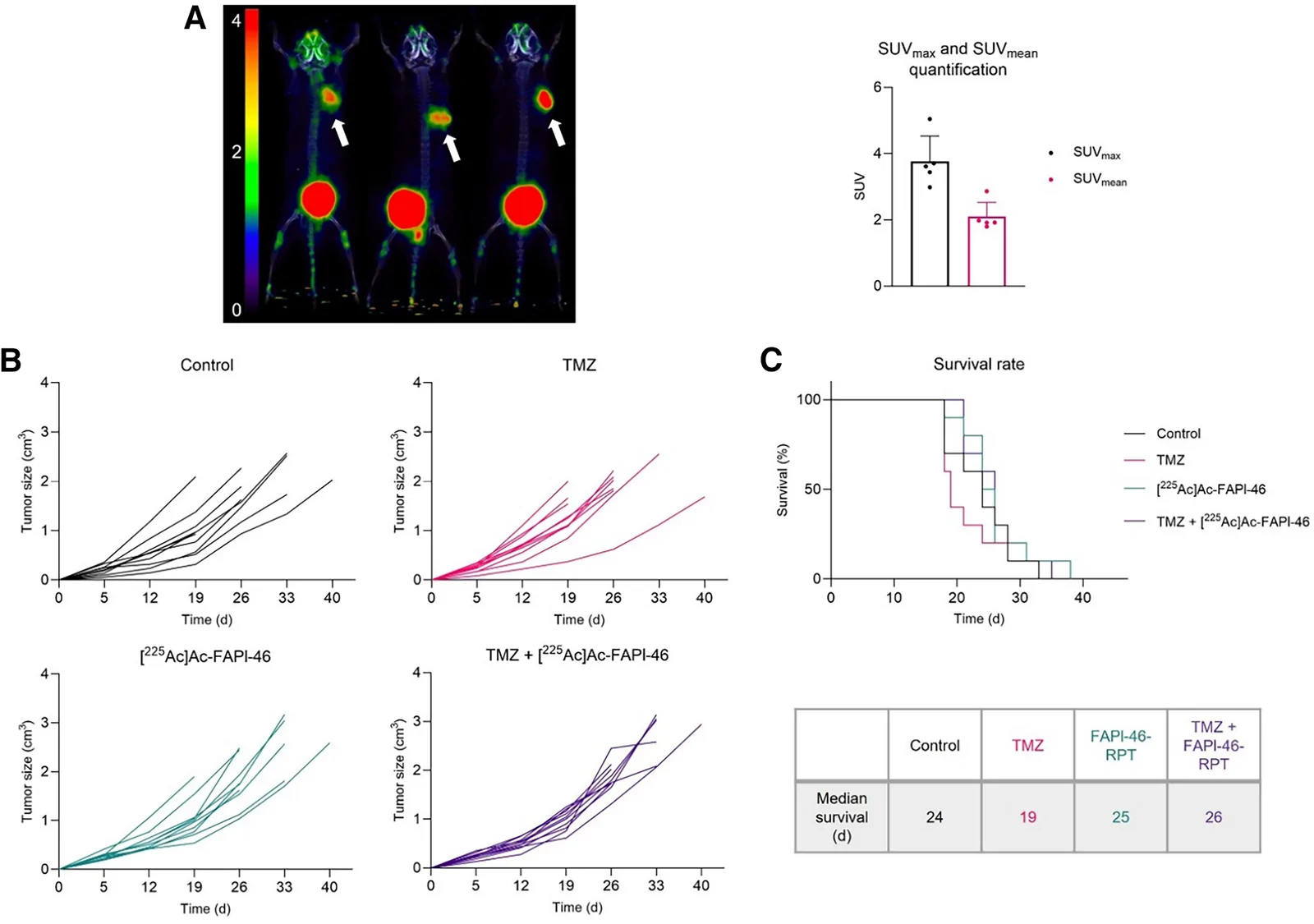
Single-dose [^225^Ac]Ac-FAPI-46 combined with TMZ in subcutaneous SB28 model. (A) [^68^Ga]Ga-FAPI-46 PET/CT in C57BL/6J mice bearing subcutaneous SB28 tumors (left), and SUV_max_ (black) and SUV_mean_ (pink) quantification (right, *n* = 4). Arrows indicate location of tumor. (B) Tumor progression in control (black), [^225^Ac]Ac-FAPI-46 (green), TMZ (pink), and combination-therapy (purple) groups (*n* = 4/group). (C) Kaplan-Meier curve and median survival table. TMZ = temozolomide.

To evaluate the therapeutic potential of [^255^Ac]Ac-FAPI-46 in an immunocompetent setting, we evaluated its efficacy in C57BL/6 mice bearing syngeneic glioblastoma. Among the SB28, CT2A, and GL261 cell lines, SB28 and CT2A exhibited in vitro sensitivity to temozolomide and radiation (Supplemental Fig. 5). SB28 was chosen for in vivo studies and demonstrated high [^68^Ga]Ga-FAPI-46 tumor uptake (SUV_max_, 3.8 ± 0.8; SUV_mean_, 2.1 ± 0.4) (Fig. 2A). However, temozolomide, single-dose [^225^Ac]Ac-FAPI-46, or their combination did not significantly affect tumor growth or median survival compared with controls (Figs. 2B and 2C).

Variations in the composition of the TME, including immune and stromal cells, can lead to distinct therapeutic outcomes (22,23). To address this, the temozolomide dose was increased to 25 mg/kg/d, and [^225^Ac]Ac-FAPI-46 (60 kBq) was administered over 3 consecutive days (Supplemental Fig. 6). Under this intensified regimen, temozolomide monotherapy at the higher dose failed to delay tumor growth or improve median survival compared with controls (33.5 vs. 35 d). In contrast, triple-dose [^225^Ac]Ac-FAPI-46 significantly delayed tumor growth and markedly prolonged median survival (60 d). The combination therapy did not enhance survival beyond [^225^Ac]Ac-FAPI-46 alone (Supplemental Fig. 6). Double-strand-break levels were significantly higher for both [^255^Ac]Ac-FAPI-46-alone and combination-therapy groups (13.9 ± 7.3% and 11.8 ± 3.0%, respectively) than for control and temozolomide groups (2.1 ± 1.1% and 2.3 ± 2.1%, respectively) (Supplemental Fig. 7). Overall, these results validate the therapeutic potential of [^225^Ac]Ac-FAPI-46 in glioblastoma, demonstrating potent DNA damage induction and survival benefit in an immunocompetent model.

### FAP RPT Induces Hematologic Toxicity

Blood toxicity was assessed by monitoring blood cell count before treatment and 24 h and 6 d post-treatment. [^255^Ac]Ac-FAPI-46 alone or with temozolomide caused significant lasting reductions in white and red blood cells and platelets, whereas temozolomide alone had milder, temporary effects (Supplemental Fig. 8).

### FAP RPT Increases Immunogenicity Without Overcoming the “Cold” TME of Glioblastoma

**FIGURE 3.**
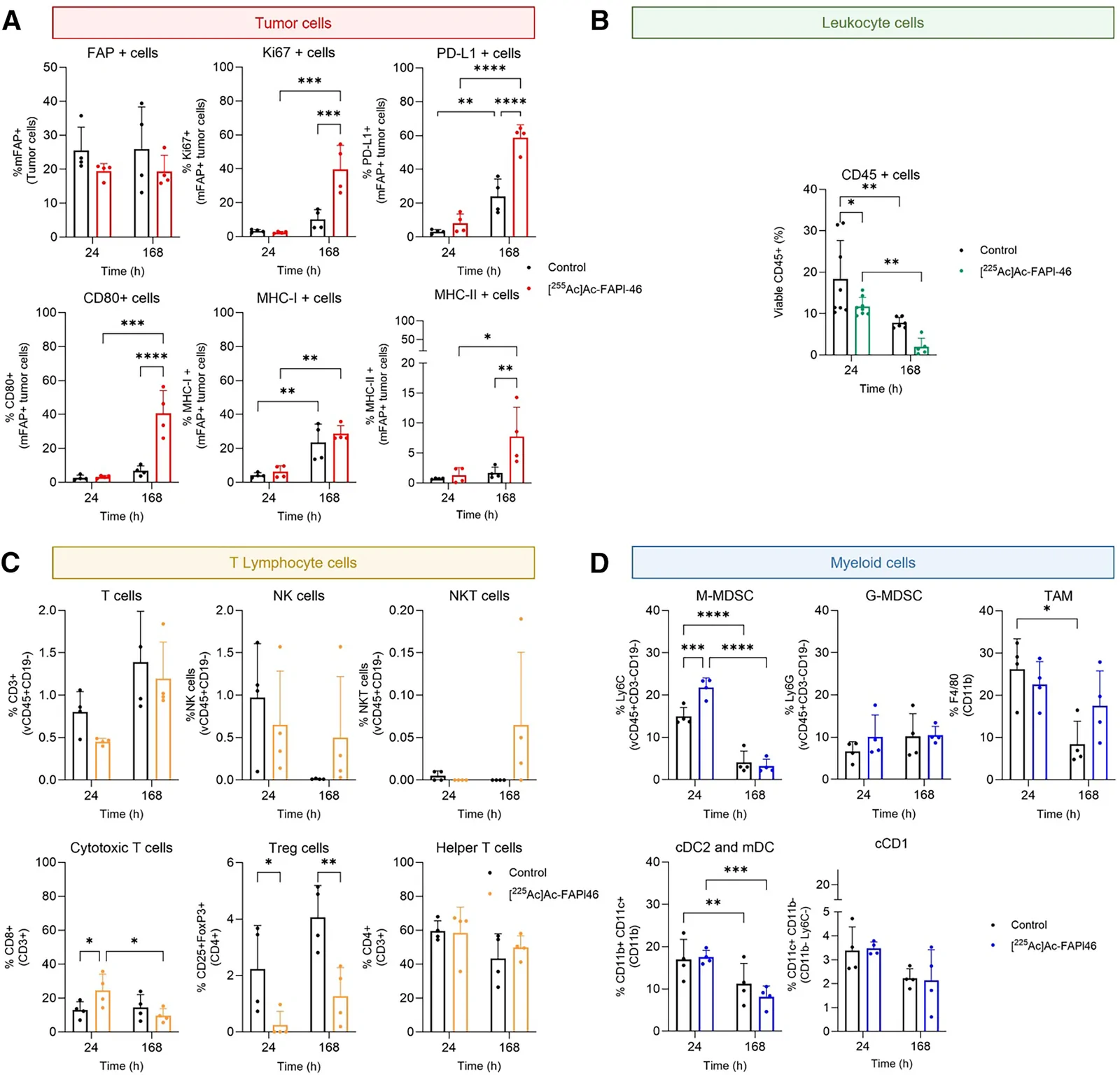
Immune profile of SB28 subcutaneous tumors after triple-dose [^255^Ac]Ac-FAPI-46 treatment. (A–D) Immune cell profile in control and [^225^Ac]Ac-FAPI-46 groups on SB28 subcutaneous tumors 24 and 168 h post-treatment (*n* = 4/group) across tumor cells (A), leukocyte cells (B), T lymphocyte cells (C), and myeloid cells (D). Statistical analyses were performed using 2-way ANOVA tests. *P < 0.05. **P < 0.01. ***P < 0.001. ****P < 0.0001. cDC1 = type 1 conventional DC; cDC2 = type 2 conventional DC; G-MDSC = granulocytic myeloid-derived suppressor cell; mDC = myeloid dendritic cell; M-MDSC = monocytic myeloid-derived suppressor cell; mFAP = murine FAP; Treg = regulatory T cells.

To characterize the immunomodulatory effects of [^255^Ac]Ac-FAPI-46, tumors were collected from control and treated mice at 24 or 168 h post-treatment, dissociated, and analyzed using spectral flow cytometry (24). [^225^Ac]Ac-FAPI-46 did not alter the proportion of murine FAP–positive tumor cells but significantly increased Ki-67 expression and reduced tumor weight at 168 h (Fig. 3A; Supplemental Fig. 9). On murine FAP–positive cells, treated tumors also exhibited significantly higher expression of CD80 and major histocompatibility complex class II (MHC-II), whereas PD-L1 and MHC-I increased in both groups at 168 h (Fig. 3A).

Within the TME, leukocytes (CD45+) significantly decreased over time (Fig. 3B). Total T cell (CD3+NK1.1−), natural killer (NK) cell (NK1.1+CD3−), and NK T cell (CD3+NK1.1+) populations were unchanged. Although T helper cells (CD3+NK1.1−CD4+CD8−) remained unaffected, regulatory T cells (CD3+NK1.1−CD4+CD25+FoxP3+) decreased significantly in treated tumors at both time points, with a transient rise in cytotoxic T cells (CD3+NK1.1−CD4−CD8+) at 24 h (Fig. 3C).

Among myeloid populations, monocytic myeloid-derived suppressor cells (CD11b+Ly6C+) significantly increased at 24 h in the treated group and then declined at 168 h in both groups, whereas granulocytes were stable (Fig. 3D). Tumor-associated macrophages (TAMs; CD11b+F4/80+) significantly decreased at 168 h in controls, M1-TAMs (CD11b+F4/80+MHC-II+CD206−CD80+CD86+) increased at 168 h in both groups, and M2-TAMs (CD11b+F4/80+MHC-II−CD206+) transiently rose at 24 h in the treated group (Supplemental Fig. 9). Monocytic dendritic cells (DCs) and type 2 conventional DCs (CD11b+DC) decreased over time, whereas type 1 conventional DCs (CD11c+CD11b−Ly6C−) remained unchanged (Fig. 3D). Fully mature DCs (CD86+CD80+) transiently increased at 24 h in treated mice, whereas immature DCs decreased at 24 h and then recovered at 168 h (Supplemental Fig. 9).

Collectively, [^255^Ac]Ac-FAPI-46 modulated immune cell infiltration in SB28 tumors but remained insufficient to convert the immunologically “cold” TME, which continued to be dominated by immunosuppressive populations. Further studies are needed to confirm and strengthen these observations.

### FAP RPT Does Not Change Overall Survival in the Orthotopic Model

**FIGURE 4.**
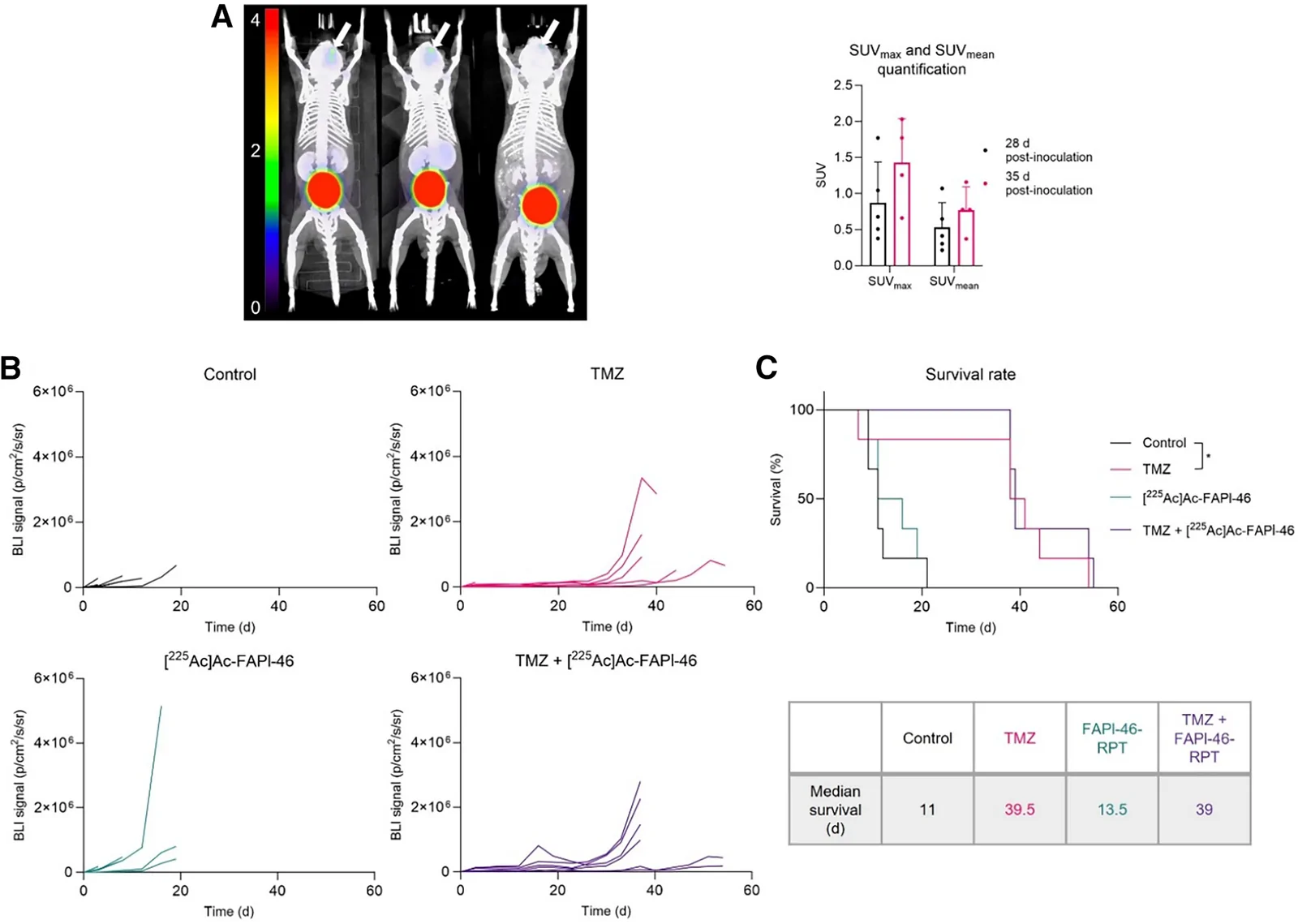
Single-dose [^225^Ac]Ac-FAPI-46 combined with TMZ in orthotopic U-87 MG model. (A) [^68^Ga]Ga-FAPI-46 PET/CT (left), and SUV_max_ (black) and SUV_mean_ (pink) quantification at 28 and 35 d post-inoculation (right, *n* = 4). Arrows indicate location of tumor. (B) Tumor progression in control (black), [^225^Ac]Ac-FAPI-46 (green), TMZ (pink), and combination-therapy (purple) groups (*n* = 5/group). (C) Kaplan-Meier curve and median survival table. Survival curves were compared using log-rank (Mantel–Cox) tests. *P < 0.05. BLI = bioluminescence imaging; TMZ = temozolomide.

In mice bearing an orthotopic U-87 MG tumor, [^68^Ga]Ga-FAPI-46 PET/CT imaging revealed low but progressively increasing tumor uptake (SUV_max_, 0.9 ± 0.6 and 1.4 ± 0.6 at 28 and 35 d, respectively; Fig. 4A). [^225^Ac]Ac-FAPI-46 monotherapy did not significantly delay tumor growth compared with controls (median survival, 11 vs. 13.5 d). In contrast, temozolomide monotherapy significantly delayed tumor growth by 28.5 d. However, combination therapy with [^225^Ac]Ac-FAPI-46 and temozolomide failed to improve survival beyond temozolomide alone (median, 39.5 d; Figs. 4B and 4C). These findings indicate that although FAPI-46 specifically targets FAP in tumors by 35 d post-implantation (SUV_max_, 1.4 ± 0.6), the uptake level is insufficient to elicit a meaningful therapeutic response.

### FAPI-46 Shows Limited Penetration Across an Intact BBB

To investigate the BBB role in governing FAPI-46 tumor accessibility, we assessed probe uptake and BBB integrity in the U-87 MG orthotopic model. At 22 d post-inoculation, neither a [^68^Ga]Ga-FAPI-46 PET signal nor Evans blue staining was detectable, indicating an intact BBB (25). By 29 d, a low but discernable PET signal emerged, although Evans blue staining remained absent, suggesting partial BBB permeability. At 36 d, both a [^68^Ga]Ga-FAPI-46 PET signal and Evans blue staining were clearly evident, with SUV_max_ values of 7.7 and 2.3, respectively. These findings suggest that the [^68^Ga]Ga-FAPI-46 PET brain signal correlates with tumor growth and progressive BBB disruption, indicating that effective delivery of [^255^Ac]Ac-FAPI-46 depends on BBB compromise (Supplemental Fig. 10).

### [^68^Ga]Ga-FAPI-46 PET/CT Shows Uptake in Patients

**FIGURE 5.**
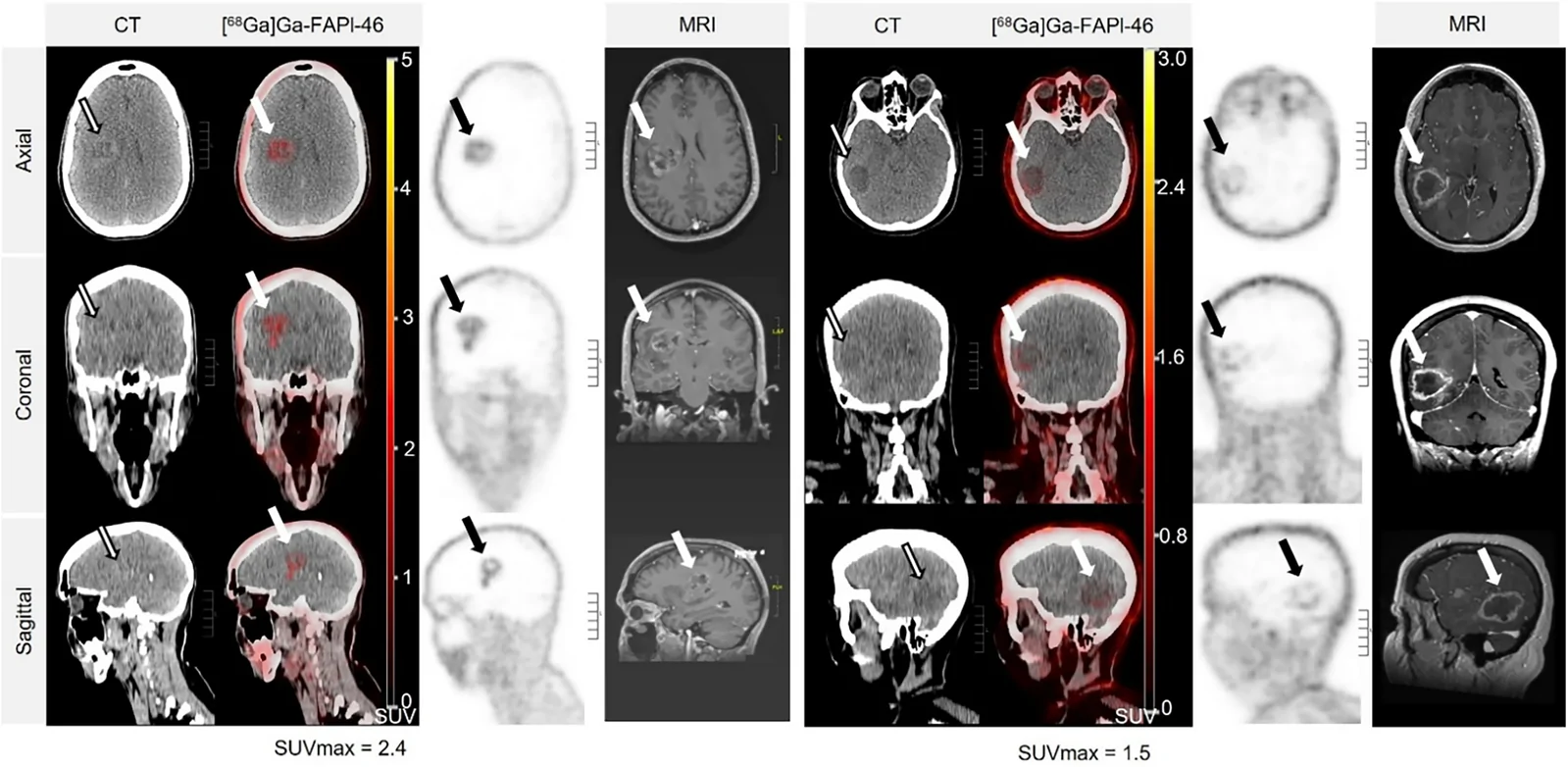
[^68^Ga]Ga-FAPI-46 PET/CT images of patients. [^68^Ga]Ga-FAPI-46 PET/CT scans were performed for 2 patients. SUV_max_ was 2.4 and 1.5. PET/CT images are shown alongside corresponding MR images. Arrows indicate location of tumor.

[^68^Ga]Ga-FAPI-46 PET/CT and 3-dimensional T1-weighted MRI were performed for 2 patients with glioblastoma: a 52-y-old man with right frontal high-grade glioma (World Health Organization grade 4, IDH wild type, and negative for IDH1 R132H mutant protein; Fig. 5) and a 46-y-old woman with right temporoparietal glioblastoma (World Health Organization grade 4, IDH-wild-type, and negative for IDH1 R132H mutant protein; Fig. 5) resected via right temporal craniotomy (12,26–28). In both cases, [^68^Ga]Ga-FAPI-46 uptake was observed at the tumor periphery, consistent with stromal or TME-associated FAP expression, and it overlapped with the MRI signal. SUV_max_ was low (1.5 and 2.4) despite the high tumor burden.

## DISCUSSION

The current glioblastoma standard of care (Stupp protocol) includes surgery, radiation, and temozolomide chemotherapy, yet the 5-y survival rate remains approximately 4% (1–5). Given these poor response rates, we subsequently evaluated FAPI-46 RPT in subcutaneous and orthotopic glioblastoma models to assess its effects on tumor cells, the immune system, and BBB-dependent treatment outcomes. This approach was supported by the low expression of FAP in normal tissues and its overexpression in glioblastoma, as confirmed by RNA sequencing of UCLA samples (10,14,18,29).

We demonstrated that [^225^Ac]Ac-FAPI-46 induced significant tumor growth delay and prolonged median survival, and it was associated with double-strand breaks in subcutaneous models. Combined with low-dose temozolomide (5 mg/kg/d), FAPI-46 RPT further delayed tumor progression. In contrast, [^177^Lu]Lu-FAPI-46 demonstrated lower therapeutic efficacy than did [^225^Ac]Ac-FAPI-46, despite 2 cycles of temozolomide, consistent with prior preclinical and clinical studies and the distinct physical properties of these radionuclides (30).

Given that immune cells make up about 30% of the TME and can influence glioblastoma treatment responses, FAPI-46 RPT was tested in the syngeneic SB28 model (2,26,27,29,30). However, unlike in the U-87 MG model, the same combination therapy did not improve outcomes. Although FAP expression was confirmed by [^68^Ga]Ga-FAPI-46 PET, SB28’s resistance (31–35) and a lower temozolomide dose likely reduced effectiveness. Variations in immune competency likely influenced the results, because doses effective in immunodeficient mice may be inadequate in immunocompetent hosts, in which both immune cells and FAP-positive stromal cells play roles in modulating treatment response (14,22,23,36).

To address these limitations, we escalated temozolomide to 25 mg/kg/d, the equivalent clinical dose of 75 mg/m^2^ (4,37), and administered FAPI-46 RPT for 3 days. Although temozolomide alone remained ineffective, triple-dose [^225^Ac]Ac-FAPI-46 reduced tumor growth and improved survival, demonstrating that α-RPT treatment intensification can overcome SB28 radioresistance.

In both models, white and red blood cell counts decreased after RPT or temozolomide, with platelet toxicity occurring after multiple RPT doses, aligning with clinical observations for RPT (38–40) and temozolomide (4,37,41). Long-term studies are needed to assess hematologic safety.

[^225^Ac]Ac-FAPI-46 showed immunomodulatory effects in the SB28 model, because it increased infiltration of cytotoxic T cells, monocytic myeloid-derived suppressor cells, and M1-TAMs while reducing immunosuppressive regulatory T cells (22,23). The low regulatory T cell percentage maintained a favorable ratio of CD8+ to regulatory T cells, highlighting FAP RPT’s immunostimulatory effect. However, M1-TAM levels remained low, and the ratio of M1-TAM to M2-TAM stayed below immune activation thresholds (42,43). Although FAPI-46 RPT upregulated immunostimulatory markers (CD80 and MHC-II) on tumor cells, the immunosuppressive marker PD-L1 remained dominant, indicating that FAPI-46 RPT alone does not overcome the immunosuppressive TME of glioblastoma.

The glioblastoma TME composition can differ substantially between subcutaneous and orthotopic models. Because FAP is expressed by both TME and tumor cells (10,13–15), evaluation of FAPI-46 RPT in an orthotopic setting was necessary. Moreover, the orthotopic model enables assessment of FAPI-46 delivery across the BBB, a major limitation for drug delivery in central nervous system malignancies (44,45). In the orthotopic U-87 MG model, PET imaging showed low tumor uptake, 4.4-fold lower than in subcutaneous tumors. Both [^225^Ac]Ac-FAPI-46 monotherapy and combination therapy did not delay tumor growth or improve overall survival compared with controls. These limited therapeutic effects are consistent with the inability of FAPI-46 to cross an intact BBB in early-stage disease and to achieve concentrations sufficient for therapy. In the preclinical glioblastoma model, early-stage PET detected no signal, whereas late-stage tumors with disrupted BBB showed increased uptake, indicating FAPI-46 cannot cross an intact BBB. Clinical PET in 2 patients confirmed low [^68^Ga]Ga-FAPI-46 uptake; an overlap between PET and MRI signals, the current standard glioblastoma imaging modality, suggests radiotracer uptake primarily in regions of BBB disruption.

Several strategies can improve BBB penetration. However, small chemical modifications are unlikely to enable passive BBB penetration of FAPI-46 because of its physicochemical profile: large molecules and high polarity (molecular weight, 885.96 g/mol) (46), low lipophilicity (log *P* = −3.57 ± 0.15 for [^68^Ga]Ga-FAPI-46), and moderate serum protein binding (52.1 ± 1.5%) (47). These properties place FAPI-46 well outside the ranges typically permissive for passive BBB diffusion: molecular weight of 400–500 g/mol, log *P* of 1–3, and high unbound plasma fraction (48). Consequently, improved FAPI-46 brain delivery will require active strategies, such as conjugation to a BBB-penetrating moiety (49,50) or combination with microbubble-assisted focused ultrasounds (51,52).

## CONCLUSION

This study demonstrates the therapeutic potential of α- and β-radiolabeled FAPI-46 in glioblastoma; however, its efficacy was model-dependent. In the aggressive immunocompetent SB28 model, FAPI-46 RPT showed limited efficacy and immune modulation, with significant survival benefit only after triple-dose [^225^Ac]Ac-FAPI-46. Limited brain penetration remains a major barrier, because both preclinical and clinical imaging revealed low tumor uptake, emphasizing the need for optimized delivery strategies to enhance BBB permeability and tumor exposure.

## DISCLOSURE

This work was supported by startup funds from the Ahmanson Translational Theranostics Division, a JCCC/DGSOM Seed Grant (UCLA CTSI, NIH UL1TR001881), and UCLA SPORE in Brain Cancer. Johannes Czernin is a founder of SOFIE Biosciences and holds equity in the company and in intellectual property invented by him, patented by the University of California, and licensed to SOFIE Biosciences. He is a founder and board member of Trethera Therapeutics and holds equity in the company and in intellectual property invented by him, patented by the University of California, and licensed to Triangle. He serves on the medical advisory board of Actinium Pharmaceuticals and on the scientific advisory boards of POINT Biopharma, RayzeBio, and Aktis Oncology. Christine Mona reports consulting activities for SOFIE Biosciences/iTheranostics unrelated to the submitted work. No other potential conflict of interest relevant to this article was reported.

## Notes

KEY POINTS **QUESTION:** Is FAP a pertinent target for RPT in glioblastoma? **PERTINENT FINDINGS:** Radiolabeled FAPI-46 demonstrated high tumor uptake and improved survival in subcutaneous glioblastoma models, particularly in combination with temozolomide. However, uptake was low in orthotopic models and in patients, consistent with limited BBB penetration. **IMPLICATIONS FOR PATIENT CARE:** Overcoming BBB limitations will be essential to translate FAPI-46–based radiotheranostics into effective clinical strategies for glioblastoma.
